# Antimicrobial Stewardship Strategies Including Point-of-Care Testing (POCT) for Pediatric Patients with Upper-Respiratory-Tract Infections in Primary Care: A Systematic Review of Economic Evaluations

**DOI:** 10.3390/antibiotics11081139

**Published:** 2022-08-22

**Authors:** Costanza Vicentini, Lorenzo Vola, Christian Previti, Valerio Brescia, Francesca Dal Mas, Carla Maria Zotti, Fabrizio Bert

**Affiliations:** 1 Department of Public Health Sciences and Pediatrics, University of Turin, 10126 Turin, Italy; 2 Department of Management, University of Turin, 10126 Turin, Italy; 3 Department of Management, Ca’ Foscari University of Venice, Cannaregio, 873, 30100 Venice, Italy

**Keywords:** children, upper-respiratory-tract infections, primary care, point-of-care tests, rapid antigen-detection tests, C-reactive protein tests, nucleic acid amplification tests, health–economic evaluations

## Abstract

Upper-respiratory-tract infections (URTIs) are among the main causes of antibiotic prescriptions in pediatric patients. Over one-third of all antibiotic prescriptions for URTIs in children are estimated to be inappropriate, as the majority of URTIs are caused by viral agents. Several strategies, including clinical scoring algorithms and different point-of-care tests (POCTs) have been developed to help discriminate bacterial from viral URTIs in the outpatient clinical setting. A systematic review of the literature was conducted following PRISMA guidelines with the objective of summarizing evidence from health–economic evaluations on the use of POCT for URTIs in pediatric outpatients. A total of 3375 records identified from four databases and other sources were screened, of which 8 met the inclusion criteria. Four studies were classified as being of high reporting quality, and three were of medium quality. Five out of eight studies concluded in favor of strategies that included POCTs, with an additional study finding several POCTs to be cost-effective compared to usual care but over an acceptable WTP threshold. This review found POCT could be a valuable tool for antimicrobial stewardship strategies targeted towards childhood URTIs in primary care.

## 1. Introduction

Acute respiratory-tract infections (RTIs) are among the main causes of antibiotic prescriptions in pediatric patients, accounting for over 70% of all antibiotics prescribed in ambulatory care in children [1,2]. Acute pharyngitis, in particular, is one of the most common reasons for which children seek primary care [3].

However, the majority of upper RTIs (URTIs) are caused by viral agents, which are most often self-limiting, and only approximately 37% of overall URTIs are caused by bacteria, of which the most common agent is group A β-hemolytic streptococcus (GAS) [4]. Over one-third of all antibiotic prescriptions for acute RTIs in children are estimated to be inappropriate [5]. The treatment of viral RTIs, in particular, accounts for an important proportion of improper antibiotic use [6], which is mostly driven by fear of severe GAS complications such as rheumatic fever, glomerulonephritis, and suppurative complications.

Considering antibiotic consumption is associated with increasing antimicrobial resistance (AMR), in addition to side effects including increased rates of *C. difficile* infection, anaphylaxis, and death, new approaches are needed to reduce diagnostic uncertainty and improve prescribing practices for RTIs [7]. Antimicrobial stewardship (AMS) programs have proven to be effective in reducing unnecessary antibiotic use and AMR rates, increasing patient safety, and reducing healthcare costs in both adult and pediatric populations [8]. However, discriminating bacterial from viral URTIs in the outpatient clinical setting is challenging.

Several strategies, including clinical scoring algorithms and different point-of-care tests (POCTs), have been developed to help discriminate between viral and bacterial agents or to allow the specific etiologic diagnosis of pharyngitis. A number of POCTs have demonstrated acceptable levels of accuracy and effectiveness [9,10]. In their systematic review and meta-analysis, Verbakel et al. found POCT in primary care reduces immediate antibiotic prescribing. However, this review focused on the clinical impact of POCTs and, in particular, on their effect on clinical decision-making [10].

Lingervelder et al. conducted a systematic review to evaluate the health–economic impact of introducing POCTs for any health condition and in any setting, concluding that, although the uptake of POCTs remains low in many countries, their implementation could be beneficial [11]. By including a broad range of indications for POCTs, the review by Lingervelder et al. provided important results. As it was beyond the remit of their review to evaluate specific outcome measures and clinical pathways, only few outcome measures were selected to evaluate the impact of POCTs so that they could be applied to all indications. A lack of evidence persists regarding the cost-effectiveness of POCTs for the diagnosis of URTIs in pediatric patients, which is necessary to support stakeholders and policymakers in the selection of the most appropriate strategies in clinical practice.

This paper aims to summarize evidence from health–economic evaluations on the use of POCTs for URTIs in pediatric outpatients. Our objective was determining (a) whether POCTs are beneficial for this indication and, if so, (b) which specific POCT is the most cost-effective and (c) which AMS approach is associated with the most favorable results from a health–economic perspective.

## 2. Materials and Methods

### 2.1. Study Design and Registration

A systematic review of the literature was conducted. A protocol for this study was registered with the PROSPERO international prospective register of systematic reviews (registration number CRD42021271601).

### 2.2. Search Strategy

The Preferred Reporting Items for Systematic Reviews and Meta-Analyses (PRISMA) guidelines were followed while carrying out this systematic review [12]. An electronic literature search for research articles published up to 9 August 2021 was performed, selecting only papers written in English. Four databases were searched: Ovid-MEDLINE, CINAHL, Embase, and Cochrane Central. Search terms included both MeSH terms and free text (keywords, synonyms, and word variations), connected with Boolean operators. Specifically, we applied “OR” in each group of keywords and MeSH terms to indicate the areas of interest and then “AND” to combine each group to find articles relating to the research question. Strings used for each database are available as Supplementary Materials.

### 2.3. Eligibility Criteria

Publications were included/excluded based on the following criteria:

- Only studies in English published after the year 2000 were included. No geographical restrictions were applied.
- Abstracts/unpublished articles were included if relevant; journal articles, commentaries, editorials, letters, (systematic) reviews, and conference abstracts were included if they reported extractable data; conference abstracts/unpublished articles or articles for which full texts were not available were excluded.
- Studies were included if a POCT was employed for diagnostic purposes, excluding the use for screenings and monitoring. Studies conducted only on adult populations were excluded. Studies without any type of economic evaluation did not meet the “cost-effectiveness and costing” eligibility criteria and were excluded from the analysis.

### 2.4. Study Selection

Screening followed a three-step process. After removing duplicates, two authors independently screened titles and abstracts for potential relevance and finally assessed the eligibility of full texts according to the previously stated inclusion/exclusion criteria. In cases of doubt about whether a publication met the criteria based on the first round of screening, the publication was included for full-text assessment. Discrepancies were resolved by consensus, and reasons for exclusion at the full-text screening phase were noted.

### 2.5. Methodological Assessment

The reporting quality of included publications was determined by evaluating how many of the 28 items contained in the 2022 version of the Consolidated Health Economic Evaluation Reporting Standards (CHEERS) checklist were met [13]. The 28 criteria items are divided according to the following sections: title and abstract (2 items), introduction (1 item), methods (18 items), results (4 items), and discussion (3 items). In the reporting-quality assessment, items that fully met the criteria defined by the checklist were given 1 point, while a score of 0 was attributed to those items that did not fully meet the criteria. Reporting quality was not considered in the inclusion or exclusion of publications; all studies were assessed for reporting quality if inclusion criteria were met.

### 2.6. Data Extraction and Management

The screening of search results was performed using the web-based, open-access platform Colandr (https://www.colandrapp.com/, accessed on 2 August 2022). Data were independently extracted by three authors into pre-defined and labeled columns in a spreadsheet.

General publication characteristics that were extracted consisted of the country where the evaluation was performed, population, setting, condition of interest, type of POCT, implementation strategy, and comparator. Further, methodological characteristics of economic evaluations were also extracted, namely whether the assessment was model- or trial-based, the type of health–economic evaluation performed, the chosen time horizon, the perspective from which the costs and effects were evaluated, and whether sensitivity analysis was performed. Outcomes of interest extracted were the impact of POCTs on costs and the impact on health outcomes, in line with the methodology employed by Lingervelder et al. [11]. Base-case results and results of sensitivity analyses, as well as the conclusions of each evaluation, were also extracted.

## 3. Results

### 3.1. Database Research

**Figure 1:**
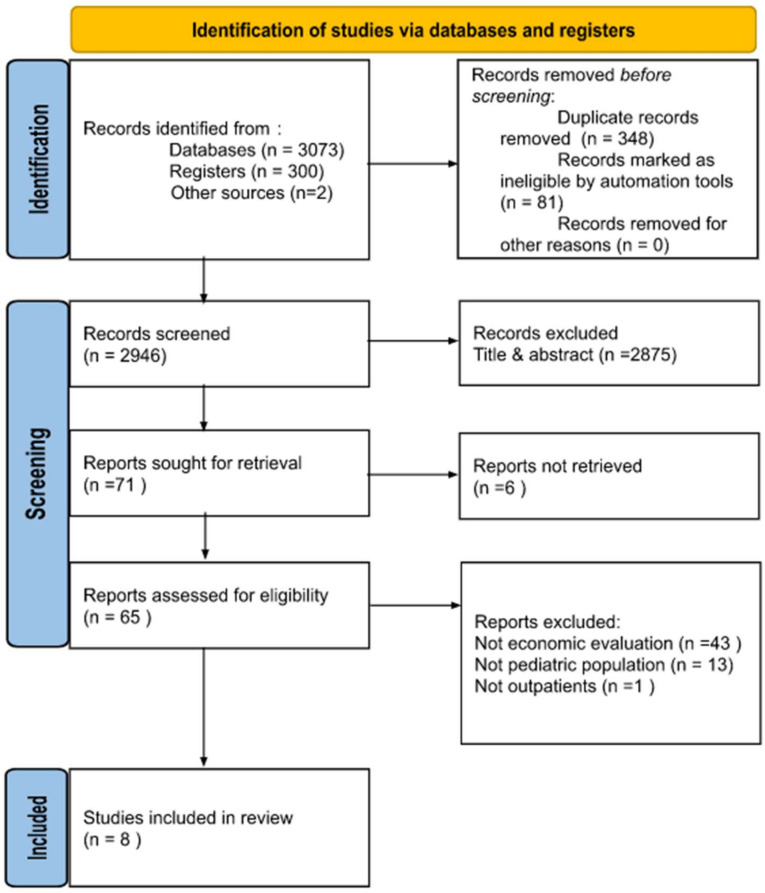
PRISMA 2020 flow diagram.

The initial search identified a total of 3073 records from databases, 300 from registers, and 2 from other sources. Prior to the screening, 429 duplicate or ineligible records were removed. A further 2875 publications were excluded following title and abstract screening, as they did not meet the eligibility criteria. Following the first stage of screening, 65 potentially relevant publications were retrieved in full text. Based on full-text assessment, 57 studies were excluded: 43 did not describe an economic evaluation, 13 studies did not include children, and 1 was not performed in a primary care setting. Ultimately, eight publications met the inclusion criteria and were included in the qualitative synthesis [14,15,16,17,18,19,20,21]. Figure 1 depicts the PRISMA flow chart.

### 3.2. Quality of Included Studies

Results of the quality assessment are reported in Table 1. Compliance with the 28 items identified by the 2022 CHEERS checklist ranged from 39.9 to 96.5%, with an average score of 21.13. Four out of eight included studies were classified as being of high reporting quality (>75%), and three were of medium quality (with a score between 50 and 75%). The items that were most frequently missed included items newly introduced to the 2022 CHEERS checklist, such as the development and availability of a health–economic-analysis plan, the approach to and effect of engagement with patients, and others affected by the study [13]. Other frequently missed items were time horizon and discount rate. The three most recent publications were among those achieving the highest scores.

### 3.3. Study Characteristics

Table 1 summarizes the characteristics of the included studies. Our search included studies published following the year 2000: two studies were published prior to 2017 and the other six between 2017 and 2021. Six studies were set in high-income countries (four in Europe and two in the US), and two were set in lower-middle-income countries (LMIC) (one in Iran and one in Vietnam).

Half of the included studies focused only on children, whereas the other half comprised both children and adults. One study evaluated both primary and secondary care, whereas the rest focused only on primary care. All studies assessed acute respiratory-tract infections (RTIs): six studies only evaluated upper RTIs (URTIs), one study evaluated both upper and lower RTIs, and one included both URTIs and otitis media.

In all included studies, POCTs were employed with a diagnostic purpose. Until 2020, the only evaluated POCTs were rapid antigen-detection tests (RADT, *n* = 5) and C-reactive protein tests (CRP, *n* = 1). The study evaluating the POC CRP was performed in a LMIC. Novel tests were evaluated from 2020 onwards by two studies: one evaluating the POC nucleic acid amplification tests (NAAT) and the other comparing standalone CRP tests, dual-biomarker tests (CRP and myxovirus-resistance protein A (MxA)), and a hypothetical test meeting a target product profile defined by the global health community in 2015 [14]. The evaluated implementation strategies for the POCTs were the standalone POCT (*n* = 7), POCT in conjunction with clinical scoring tools (e.g., Centor and FeverPAIN score, *n* = 3), POCT with throat-swab-culture confirmation of negative results (n = 4), and POCT in conjunction with culture (*n* = 1). Non-POCT comparators included routine clinical practice (clinical examination, *n* = 3), observation (no treatment, *n* = 2), treatment with antibiotics of all suspected cases (*n* = 3), culture (*n* = 4), and use of clinical scoring tools (*n* = 3).

### 3.4. Methodological Characteristics of Economic Evaluations

Table 2 reports the methodological characteristics of economic evaluations reported by the included studies. Three studies were cost–utility analysis, one was a budget–impact analysis, one was both a cost–utility and budget–impact analysis, and the remaining three were, separately, a cost–benefit analysis, a cost–effectiveness analysis, and a cost–identification analysis. The type of economic evaluation was stated in the title of five studies. The majority of studies were model-based (*n* = 6), five of which used decision-tree models. Concerning the two trial-based studies, the number of included patients was, separately, 72 and 2037.

The selected time horizon ranged from 14 days to 5 years, with 1 year selected by three economic evaluations. Four studies did not report the selected time horizon. The most frequently chosen perspective was the healthcare-system perspective (*n* = 4), followed by societal (*n* = 3) and payer (*n* = 3), with two studies using both societal and payer perspectives.

Outcomes were reported in terms of cost per patient (*n* = 4), cost–effectiveness (*n* = 5), incremental-cost–effectiveness ratio (ICER, *n* = 4), and total cost (*n* = 3). Five studies performed deterministic-sensitivity analyses (DSA), one performed probabilistic-sensitivity analyses (PSA), and one performed both DSA and PSA. Both studies including PSA were decision-tree models published after 2020. One trial-based study did not assess uncertainty.

### 3.5. Economic-Evaluations Results

The results of the included studies are summarized in Table 2. POCT costs ranged from USD 0.84 to 16.53 for RADT and USD 3 to 17.8 for POC CRP, whereas estimated costs for dual-biomarker tests and POC NAAT were USD 14.83 and 25.65, respectively. Five studies concluded in favor of POCTs, whereas two studies concluded in favor of usual care. Considering the investigated implementation strategies, standalone POCT was the preferred strategy according to four studies: RADT in two studies, POC NAAT in one study, and, in the study investigating a hypothetical test, CRP and MxA, and CRP, all were found to be beneficial compared to usual care. RADT in conjunction with a clinical scoring tool was found to be the most cost-effective strategy by one study. However, the same study found that standalone RADT was the most cost-effective strategy when the sensitivity and specificity of the clinical score decreased. No study found POCT with culture confirmation of negative results to be the preferred strategy. However, one study found POCT in conjunction with culture or culture alone to be the most cost-effective strategies in some simulations, depending on peritonsillar abscess probability. Another study found culture alone to be the preferred strategy considering Medicaid reimbursements.

Considering the two studies that found usual care to be the preferred strategy, one found CRP would be cost-beneficial if adherence to test results increased. The other study found 17 out of 21 investigated tests were cost-effective compared to usual care but over the willingness-to-pay (WTP) threshold.

## 4. Discussion

Children are high consumers of antibiotics, which are globally the most commonly prescribed therapeutic agents in the pediatric population. Over 50% of pediatric inpatients are estimated to receive antibiotics, with more than a third of antimicrobials prescribed for the treatment of community-acquired infections [22,23,24].

An important proportion of antimicrobial prescriptions in children are estimated to be unnecessary or inappropriate [5]. According to an analysis of GP-prescribing practices in the UK in 2018, nearly half of RTI consultations lead to antibiotic prescription. The proportion of appropriate prescriptions was 0% for common cold and flu-like illness, 11% for acute rhinosinusitis, 13% for acute sore throat, and 17% for acute otitis media. The most frequently prescribed agents for URTIs were amoxicillin, penicillin-V, and erythromycin [14].

Promoting the appropriate use of antibiotics is a recognized patient-safety and public-health priority, as unnecessary treatments increase the risk of adverse events, including anaphylaxis and death, increase community-acquired *Clostridium difficile* infections, and raise healthcare costs. Further, antibiotic overuse is considered among the most important contributing factors to the significant challenge posed by AMR [8,22,25].

A growing body of literature has focused on AMS programs in pediatric populations; however, increased efforts are needed, in particular, concerning the outpatient setting [22]. Diagnostic testing plays a critical role in supporting clinical decision-making and is an essential component of AMS strategies [26]. POCTs, in particular, have the potential of significantly improving healthcare delivery in primary care, as they can be performed directly during consultations, with results available in minutes, allowing timely and appropriate treatment decisions to be made [11].

On the other hand, introducing POCTs could increase costs, as well as labor and competency requirements [11]. Therefore, comparing the benefits of POCTs and the related costs and efforts required by the clinical staff is worth investigating. Further, accurately diagnosing URTIs is important, as untreated GAS infections can lead to severe sequelae, such as acute rheumatic fever and glomerulonephritis, as well as suppurative complications, including peritonsillar abscess, mastoiditis, and cervical lymphadenitis. Antibiotic treatment of GAS URTIs decreases symptom duration and clinical severity and contributes to reducing person-to-person transmission [9]. It is, therefore, necessary to determine if the benefits of POCTs outweigh burdens.

Currently, there is a lack of consensus regarding the best strategy to manage URTIs in children. This review summarized evidence from eight health–economic evaluations of the use of POCTs for URTIs in pediatric outpatients, aiming to determine the most cost-effective management strategy. Even though the number of included studies was relatively small, the majority of publications were of moderate-to-high reporting quality, with over half classified as being of high quality. Among the items that were most frequently missed were items introduced in the 2022 version of the CHEERS checklist. Among these items, there are the development and availability of a health–economic-analysis plan, the approach to and effect of engagement with patients and others affected by the study [13], which should be considered, as all studies included in this review were published prior to 2022. In any case, even though this review focused on a specific indication for POCTs, the included studies were set in different countries with very different healthcare systems and reimbursement methods. Therefore, some caution should be applied when comparing results.

Five out of eight studies concluded in favor of strategies that included POCTs, with an additional study finding several POCTs to be cost-effective compared to usual care but over an acceptable WTP threshold [14,15,16,19,21]. Of six studies evaluating strategies including RADT, three found strategies including RADT to be the most beneficial [15,16,19]. One study directly compared a strategy combining RADT and culture to standalone POC NAAT, finding in favor of the latter [21]. Two studies evaluated strategies including POC CRP, with one directly comparing standalone POC CRP and dual-biomarker tests (CRP and MxA) and concluding the latter was the most cost-saving [14,18]. No studies directly compared RADT and POC CRP, nor POC CRP and POC NAAT.

**Figure 2:**
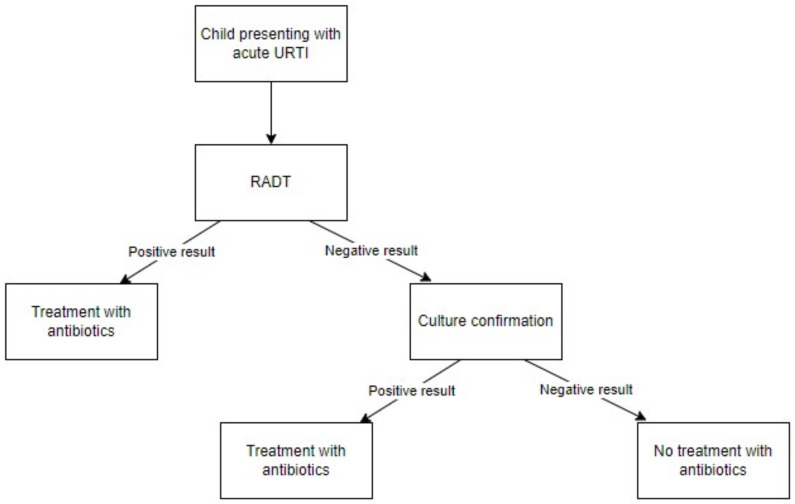
Flow chart summarizing the 2012 Infectious Diseases Society of America (IDSA) guidelines for managing group A streptococcal pharyngitis (GAS) [28]. RADT: rapid antigen-detection test; URTI: upper-respiratory-tract infection.

According to the most recent Infectious Diseases Society of America (IDSA) recommendations, which were updated in 2012, RADT or bacterial culture should be employed for children over three years of age presenting suspected GAS URTI; due to the high specificity but varying sensitivity of RADTs, if RADTs are employed, negative results should be confirmed with culture [27]. This strategy allows the identification of nearly all GAS infections but involves a second round of testing for patients with non-GAS URTIs, which requires the specimen to be sent to a laboratory for analysis and at least two days to return results [21]. A flow chart summarizing IDSA recommendations is presented in Figure 2.

**Figure 3:**
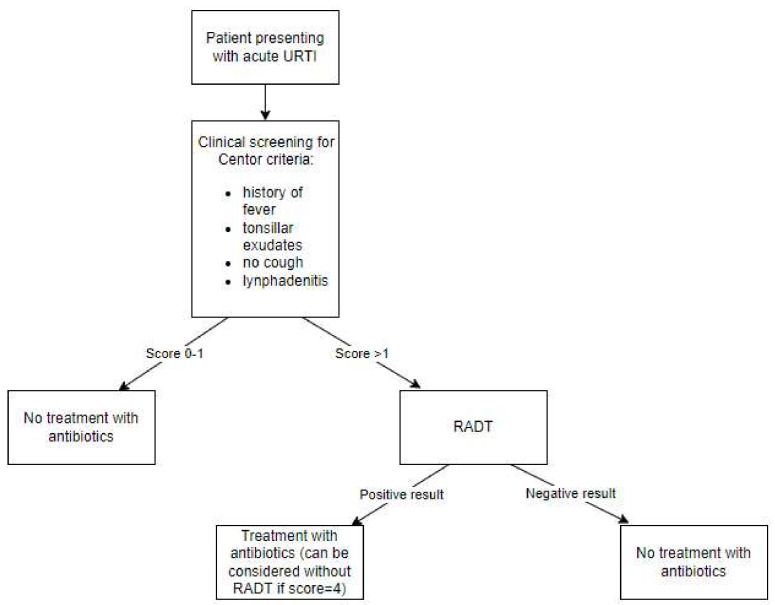
Flow chart summarizing the American Academy of Family Physicians (Leawood, KS, USA) guidelines for managing group A streptococcal pharyngitis (GAS) using a clinical scoring tool in combination with a rapid antigen-detection test (RADT) [29]. URTI: upper-respiratory-tract infection.

Interestingly, none of the four studies included in this review that evaluated this approach found it to be the most cost-effective strategy [15,16,19,21]. One study found using a clinical scoring tool in combination with RADT, which is the approach recommended by the American Academy of Family Physicians and the Centers for Disease Control (Leawood, KS, USA) for adult patients, to be the most favorable strategy, even compared to the strategy recommended by the IDSA [16,29]. The approach aims to increase the sensitivity of RADT and consists of a three-step process, which involves triaging patients using a clinical scoring system, performing RADT on patients with high scores, and treating patients with a positive result [16]. Figure 3 summarizes this strategy.

Several biomarkers have been evaluated in the context of URTIs in primary care, such as POC CRP and MxA. Both are markers of the host’s systemic immune response to clinical infection. CRP is a non-specific inflammatory marker, whereas MxA is specific for viral infections. POC CRP has demonstrated high discriminatory power in distinguishing viral from bacterial infections and can be combined with MxA to increase sensitivity, specificity, and overall accuracy [14,28].

Two recent systematic reviews found introducing POC CRP in primary care significantly reduced the rate of antibiotic prescribing when clinical guidance on the use and interpretation of POC CRP cutoffs was provided, for pediatric patients in particular [10,28]. Guideline adherence could be an important factor to consider in the implementation of POCTs. Of the two studies included in this review evaluating POC CRP, one found usual care was the most cost-beneficial strategy; however, results of the sensitivity analysis led the authors to conclude that test adherence was critical in ensuring the cost-effectiveness of POC CRP [18].

Results of previous studies also suggest interventions combining POC CRP and training are more effective in reducing antibiotic prescriptions for RTIs in primary care [10,30,31], with one randomized controlled trial finding a combined intervention of CRP guidance and enhanced communication-skills training to be associated with the greatest reduction in prescribing rate [31]. These findings suggest a broader AMS strategy combining POCT with training and education initiatives targeted toward both general practitioners and patients should be considered to increase effectiveness when introducing POC CRP in primary care [32]. An economic evaluation of an intervention combining POC CRP and communication-skills training found the strategy to be cost-effective in reducing antibiotic prescriptions for lower RTIs in general practice at a very low WTP [30]. The cost-effectiveness of this approach for URTIs in children remains to be determined, as no study included in this review evaluated training or education.

Recently, POC NAAT have been developed for the detection of GAS. Previous studies suggest their accuracy could be superior to RADT with culture confirmation of negative results [33]. As the sensitivity of these tests is higher compared to RADT, culture confirmation is not required according to test manufacturers, significantly reducing turn-around times. Further, optical readers are available to interpret results, reducing interobserver variability and subjectivity compared to RADT [9]. Even though the cost of POC NAAT is significantly higher than that of RADT, due to more expensive reagents and instrumentation, one study included in this review that directly compared POC NAAT to RADT and culture still found the benefits of POC NAAT in terms of reduced GAS complications outweighed increased costs [21]. It must be noted that the considerations regarding training and education particularly apply to POC NAAT, as issues exist, such as specimen collection, environmental contamination, and nucleic acid contamination of assays [9].

Several implementation strategies have been proposed for pediatric AMS strategies in the outpatient setting [8]. Developing local evidence-based clinical practice guidelines, as well as antibiotic handbooks providing information on appropriate dosing, duration, formulation, and alternatives for patients allergic to penicillin, have both proven effective [34]. The most appropriate diagnostic stewardship approach including POCT could be incorporated into these booklets and guidelines. Concerning educational interventions for frontline practitioners, approaches should also integrate parent expectations, with the objective of developing partnerships with all stakeholders [34].

Other than the previously mentioned limitations to this study, other aspects should be considered when interpreting results. As we performed our search on four databases, not all economic evaluations may have been identified. We chose not to include studies published prior to 2000, as the sensitivity of POCT, and of RADT in particular, could be inconsistent with today’s clinical practice [15]. All results of model-based evaluations are limited by the validity of the authors’ assumptions. Finally, several studies failed to report the considered time frame for the economic assessment. Future health–economic evaluations of POCT in this context should adopt similar methodologies to increase comparability.

## 5. Conclusions

Despite its limitations, this review found POCT could be a valuable tool for AMS strategies targeted towards childhood URTIs in primary care. POC NAAT appears a promising tool; however, further studies directly comparing POC NAAT to other cost-effective strategies, such as POC CRP and dual-biomarker tests, are warranted. Nevertheless, as previously stated, the studies included in this review were setting-specific, which could limit the generalizability of results.

The decision on whether to prescribe antibiotics is a complex process, in which doctors are influenced not only by diagnostic uncertainty but also by patient concerns and expectations, time constraints, and externalized responsibility. Even though the evidence provided by this review supports the role of POCT in this context, diagnostic tools should be incorporated into broader AMS strategies addressing the full range of drivers of inappropriate antibiotic prescribing [22]. Further health–economic evidence will be useful to guide the implementation of more comprehensive AMS strategies, such as multifaceted interventions combining POCT, training, and education. Finally, the development of new technologies for POCT will require further evaluations of costs and benefits.

## Figures and Tables

**Table 1:** Characteristics of studies reporting economic evaluations of antimicrobial stewardship strategies including point-of-care testing in pediatric patients with respiratory-tract infections.

First Author, Year of Publication	Country	Population	Setting	Condition	POCT	Implementation Strategy for POCT	Non-POCT Comparator	Study Quality (CHEERS Score)
Van Howe, 2006 [15]	USA	Children (age not specified)	Primary care	URTI	RADT	(1) Standalone RADT, (2) RADT with culture confirmation of negative results, (3) clinical scoring tool + RADT	(1) No treatment, (2) treat all suspected cases, (3) perform culture	21
Giraldez-Garcia, 2011 [16]	Spain	Children (aged 2–14 years)	Primary care	URTI	RADT	(1) Standalone RADT, (2) RADT with culture confirmation of negative results, (3) clinical scoring tool + RADT	(1) Treat all suspected cases, (2) perform culture, (3) clinical scoring tool	22
Malecki, 2017 [17]	Poland	Children (aged 2–15 years)	Primary healthcare centers	URTI	RADT	Standalone RADT	Routine clinical practice	11
Lubell, 2018 [18]	Vietnam	Both children and adults (aged 1–65 years)	Primary healthcare centers	RTI	CRP	Standalone POC CRP	Routine clinical practice	21
Behnamfar, 2019 [19]	Iran	Children (aged 4–12.5 years)	Outpatient (GPs, pediatricians)	URTI	RADT	(1) Standalone RADT, (2) RADT plus culture, (3) RADT with culture confirmation of negative results	(1) Treat all suspected cases, (2) no treatment, (3) perform culture	21
Fraser, 2020 [20]	UK	Both children (aged 5–14 years) and adults (aged 15–75, modeled separately)	Primary and secondary care (modeled separately)	URTI	17 different RADTs and four molecular tests	POCT + clinical scoring tool	Clinical assessment incorporating clinical scoring tools	27
Schneider, 2020 [14]	UK	Both children and adults (age not specified)	Outpatient	URTI and otitis media	CRP, dual-biomarker (CRP and MxA), hypothetical test	Standalone POCT	Routine clinical practice	22
Bilir, 2021 [21]	USA	Both children (<18 years) and adults (≥18 years)	Ambulatory care	URTI	NAAT vs. RADT	(1) Standalone POCT NAAT, (2) RADT with culture confirmation of negative results	(Only in budget impact analysis) All diagnostic techniques available in the USA (including culture and clinical scoring tools)	24

CRP: C-reactive protein; MxA: myxovirus-resistance protein A; NAAT: POC nucleic acid amplification test; POCT: point-of-care test; RADT: rapid antigen-detection test; URTI: upper-respiratory-tract infection.

**Table 2:** Methodological characteristics and results of economic evaluations of antimicrobial stewardship strategies including point-of-care testing in pediatric patients with respiratory-tract infections.

First Author, Year of Publication	Study Characteristics						Results	
	Study Design	Model-Based vs. Trial-Based	Time Horizon	Perspective	Outcomes	Sensitivity Analysis	Base-Case Results	Sensitivity-Analysis Results
Van Howe, 2006 [15]	CUA	Model-based (decision tree)	NR	Societal, payer	Cost–effectiveness	DSA	RADT had the best cost–utility result from the payer perspective.	Considerable overlap among all of the options except (1) treating all patients and (2) observing all patients.
Giraldez-Garcia, 2011 [16]	CEA	Model-based (decision tree)	NR	Healthcare system	Cost–effectiveness, ICER, total annual cost	DSA	RADT combined with clinical score was the most cost-effective strategy.	Standalone RADT was the most cost-effective strategy when the sensitivity and specificity of clinical score decreased.
Malecki, 2017 [17]	CIA	Trial-based	NR	Healthcare system	Cost per patient	Not performed	Threshold cost per test set at PLN 12	Not performed
Lubell, 2018 [18]	CBA	Trial-based	14 days	Societal	Cost per patient	DSA	CRP testing was not cost-beneficial compared to usual care.	If adherence to test result increased, POCT would be cost-beneficial.
Behnamfar, 2019 [19]	CUA	Model-based (decision tree)	NR	Societal, payer	Cost–effectiveness, ICER	DSA	RADT was the most cost-effective strategy.	(1) RADT + culture and (2) culture were the most cost-effective strategies in some scenarios (varying the probability of peritonsillar abscess).
Fraser, 2020 [20]	CUA	Model-based (decision tree)	1 year	Healthcare system and Personal Social Services perspective	Cost–effectiveness, ICER	PSA	Children’s primary care model: usual care was dominant compared to 4 tests; the other 17 tests were cost-effective compared to usual care but over WTP.	In line with deterministic results.
Schneider, 2020 [14]	BIA	Model-based	1 year	Healthcare system	Cost per patient, total annual cost	DSA	All POCTs were cost-saving compared to status quo: hypothetical test −54%, CRP + MxA −27%, and CRP −11%.	Confirmed usual care to be the highest-cost prescription strategy, followed by CRP.
Bilir, 2021 [21]	CUA, BIA	Model-based (decision tree)	CUA: 1 year, BIA: 5 years	Payer, third-party payer	Cost per patient, cost–effectiveness, ICER, total costs over 5 years	DSA and PSA	POC NAAT was dominant compared to RADT + culture.	POC NAAT remained cost-saving across all simulations.

BIA: budget–impact analysis; CBA: cost–benefit analysis; CEA: cost–effectiveness analysis; CIA: cost–identification analysis; CUA: cost–utility analysis; DSA: deterministic-sensitivity analysis; ICER: incremental-cost–effectiveness ratio; NR: not reported; PSA: probabilistic-sensitivity analysis; WTP: willingness to pay.

## Data Availability

Not applicable.

## Supplementary Materials

The following supporting information can be downloaded at https://www.mdpi.com/article/10.3390/antibiotics11081139/s1, File S1: search strategy.
