# Supplementary material for: Antimicrobial Stewardship Strategies Including Point-of-Care Testing (POCT) for Pediatric Patients with Upper-Respiratory-Tract Infections in Primary Care: A Systematic Review of Economic Evaluations
Source: Antibiotics (Basel). 2022 Aug 22;11(8):1139. doi: 10.3390/antibiotics11081139 (PMC9404955; doi:10.3390/antibiotics11081139)
Supplement: Supplementary file 1 [file antibiotics-11-01139-s001.zip › antibiotics-1861715-supplementary.pdf]

Search strategy

Cochrane central
Date Run: 27/07/2021 13:05:14

ID	Search	Hits
#1	pharyngitis OR tonsillitis OR tonsillopharyngitis OR pharyngotonsillitis OR nasopharyngitis	5010
#2	sore* NEAR/2 throat*	3445
#3	(throat* or pharyn* or tonsil*) NEAR/4 (infect* or inflam* or strep*)	1316
#4	pharynx	1686
#5	"group a" near/5 streptococc*	502
#6	gabhs OR beta-hemoly* or beta-haemoly* OR lancefield group a OR streptococcus pyogenes or "s. pyogenes" or "s.pyogenes"	734
#7	streptococc* NEAR/4 infect*	1422
#8	#1 OR #2 OR #3 OR #4 OR #5 OR #6 OR #7	10648
#9	(econom* or cost* or utilit* or benefit* or allocat* or effective*)	559809
#10	immunoassay* OR immunoenzyme technique*	4086
#11	enzyme near/2 (immunoassay* or immuno-assay* or immunosorbent)	10344
#12	immunochromatograph* OR immunosorbent technique* OR enzyme-linked immunosorbent assay* OR elisa OR elisas OR eia OR eias	15431
#13	sandwich* near/2 assay*	36
#14	lateral flow near/2 assay	35
#15	optical near/2 (immunoassay* or immuno-assay*)	9
#16	oia or oias OR bacterial antigen* OR diagnostic reagent kit OR diagnostic reagent kits OR point-of-care systems	4436
#17	(rapid or "point of care" or "near patient" or poc or poct or bedside) near/5 (test or tests or testing or detect* or diagnos* or screen* or kit or kits or assay*)	5067
#18	radt or radts or rdt or rdts	455
#19	antigen* near/3 detect*	597
#20	c-reactive protein OR crp	27267
#21	#10 OR #11 OR #12 OR #13 OR #14 OR #15 OR #16 OR #17 OR #18 OR #19 OR #20	51471
#22	#8 AND #9 AND #21 in Trials (Word variations have been searched)	300

Date: July 27, 2021

Ovid MEDLINE(R) and Epub Ahead of Print, In-Process, In-Data-Review & Other Non-Indexed Citations, Daily and Versions(R) <1946 to July 26, 2021>

1 pharyngitis.mp. or exp Pharyngitis/ 18814
2 tonsillitis.mp. [mp=title, abstract, original title, name of substance word, subject heading word, floating sub-heading word, keyword heading word, organism supplementary concept word, protocol supplementary concept word, rare disease supplementary concept word, unique identifier, synonyms] 9377
3 (tonsillopharyngitis or pharyngotonsillitis or nasopharyngitis).mp. [mp=title, abstract, original title, name of substance word, subject heading word, floating sub-heading word, keyword heading word, organism supplementary concept word, protocol supplementary concept word, rare disease supplementary concept word, unique identifier, synonyms] 2140
4 (sore* adj2 throat*).mp. [mp=title, abstract, original title, name of substance word, subject heading word, floating sub-heading word, keyword heading word, organism supplementary concept word, protocol supplementary concept word, rare disease supplementary concept word, unique identifier, synonyms] 6253
5 ((throat* or pharyn* or tonsil*) adj4 (infect* or inflam* or strep*)).mp. [mp=title, abstract, original title, name of substance word, subject heading word, floating sub-heading word, keyword heading word, organism supplementary concept word, protocol supplementary concept word, rare disease supplementary concept word, unique identifier, synonyms] 7193
6 Pharynx/mi 3790
7 ("group a" adj5 streptococc*).mp. [mp=title, abstract, original title, name of substance word, subject heading word, floating sub-heading word, keyword heading word, organism supplementary concept word, protocol supplementary concept word, rare disease supplementary concept word, unique identifier, synonyms] 10331
8 gabhs.mp. [mp=title, abstract, original title, name of substance word, subject heading word, floating sub-heading word, keyword heading word, organism supplementary concept word, protocol supplementary concept word, rare disease supplementary concept word, unique identifier, synonyms] 405
9 (beta-hemoly* or beta-haemoly*).mp. [mp=title, abstract, original title, name of substance word, subject heading word, floating sub-heading word, keyword heading word, organism supplementary concept word, protocol supplementary concept word, rare disease supplementary concept word, unique identifier, synonyms] 5595
10 lancefield group a.mp. [mp=title, abstract, original title, name of substance word, subject heading word, floating sub-heading word, keyword heading word, organism supplementary concept word, protocol supplementary concept word, rare disease supplementary concept word, unique identifier, synonyms] 133
11 Streptococcus pyogenes/ 13836
12 (streptococcus pyogenes or "s. pyogenes" or "s.pyogenes").mp. [mp=title, abstract, original title, name of substance word, subject heading word, floating sub-heading word, keyword heading word, organism supplementary concept word, protocol supplementary concept word, rare disease supplementary concept word, unique identifier, synonyms] 18089
13 Streptococcal Infections/ 33863

Embase®

Embase Session Results

Date: July 27, 2021

No.	Query	Results
#40	#39 AND [embase]/lim	2,275
#39	#15 AND #18 AND #38	2,425
#38	#19 OR #20 OR #21 OR #22 OR #23 OR #24 OR #25 OR #26 OR #27 OR #28 OR #29 OR #30 OR #31 OR #32 OR #33 OR #34 OR #35 OR #36 OR #37	1,248,288
#37	crp	109,034

Accessibility Information and Tips

Print Search History

Tuesday, July 27, 2021 10:27:22 AM

#	Query	Limiters/Expanders	Last Run Via	Results
S41	S14 AND S19 AND S39	Limiters - Exclude MEDLINE records<br>Expanders - Apply equivalent subjects<br>Search modes - Boolean/Phrase	Interface - EBSCOhost Research Databases<br>Search Screen - Advanced Search<br>Database - CINAHL Complete	83
S40	S14 AND S19 AND S39	Expanders - Apply equivalent subjects<br>Search modes - Boolean/Phrase	Interface - EBSCOhost Research Databases<br>Search Screen - Advanced Search<br>Database - CINAHL Complete	314
S39	S20 OR S21 OR S22 OR S23 OR S24 OR S25 OR S26 OR S27 OR S28 OR S29 OR S30 OR S31 OR S32 OR S33 OR S34 OR S35 OR S36 OR S37 OR S38	Expanders - Apply equivalent subjects<br>Search modes - Boolean/Phrase	Interface - EBSCOhost Research Databases<br>Search Screen - Advanced Search<br>Database - CINAHL Complete	186,846
S38	crp	Expanders - Apply equivalent subjects<br>Search modes - Boolean/Phrase	Interface - EBSCOhost Research Databases<br>Search Screen - Advanced Search<br>Database - CINAHL Complete	14,717
S37	(MH "C-Reactive Protein")	Expanders - Apply equivalent subjects<br>Search modes - Boolean/Phrase	Interface - EBSCOhost Research Databases<br>Search Screen - Advanced Search<br>Database - CINAHL Complete	17,668
S36	antigen* n3 detect*	Expanders - Apply equivalent subjects<br>Search modes - Boolean/Phrase	Interface - EBSCOhost Research Databases<br>Search Screen - Advanced Search<br>Database - CINAHL Complete	1,422
